# PReS-FINAL-2355: Comparison of pediatric and adult SLE genetic load

**DOI:** 10.1186/1546-0096-11-S2-P345

**Published:** 2013-12-05

**Authors:** MD Dominguez, ED Silverman, J Beyene

**Affiliations:** 1 Rheumatology, Hospital for Sick Children, Toronto, Canada; 2 Bioestatistics, McMaster University, Hamilton, Canada

## Introduction

Systemic Lupus Erythematosus (SLE) is a chronic, multisystem autoimmune disease that is the result of the interaction between genetic and environmental factors. Specific genes have been found to be related to the development of SLE and also single nucleotide polymorphisms (SNPs) have been associated to this condition. Although patients with pediatric-onset SLE (pSLE) and adult-onset SLE (aSLE) differ in disease presentation, activity, and outcomes, all studies to date have not found any specific or unique set of genes in pSLE compared to aSLE. Moreover, it has been shown that the frequency of genes in these two SLE populations is the same. However, is possible that an increased genetic load may explain the earlier onset and more severe disease seen in pSLE as compared to aSLE patients.

## Objectives

· To determine if patients with pSLE are more likely to have higher genetic load of SNP's associated with SLE than patients with aSLE · To investigate if this genetic load follows an age-related gradient.

· To define if the higher genetic load in pSLE is associated with more severe disease in this population

## Methods

**study design and patients: **This is a cross-sectional study of approximately 1000 pSLE and1000 aSLE patients. Adult and pediatric cohorts were obtained from centers in USA and Canada.

**Inclusion criteria: **SLE patients who are followed at the participating centers and who meet at least 4 of 11 ACR classification criteria for this disease are eligible for study.

## Variables

Disease severity will be measured by major organ involvement and damage (Systemic Lupus International Collaborating Clinics/ACR Damage Index (SLICC-DI))

## Genotyping

We collected DNA from the collaborating centers. The DNA will be isolated from blood samples. Genetic polymorphisms will be determined using the following techniques: ***a)Immunochip array***: an Illumina Infinium genotyping chip, containing 195,806 SNPs associated with the major autoimmune diseases including SLE. ***b) SLE designed Illumina GoldenGate Genotyping Assay: ***a flexible, pre-optimized assay that uses a discriminatory DNA polymerase and ligase to interrogate from 384 to 3,072, SNP loci simultaneously. Approximately 162 SLE susceptibility genes will be tagged with these techniques

## Results

We will determine a genetic score for each patient as explained as follow:

1. Simple count: protective SNPs and susceptibility SNPs

2. Total count: susceptibility SNPs-protective SNPs

3. Susceptibility score (SS): summation of the relative risk (RR) of each susceptibility SNP

4. Protective score (PS): 1 - RR of each protective SNP

5. Summation core (SMS): SS - PS

After performing the descriptive analysis, we will determine if the genetic load is higher in pediatric as compared with adult populations and whether there is an age-related gradient of the genetic load within and between these populations. In addition, we will determine if the genetic load is related to disease severity and damage in these populations individually and for the complete adult and pediatric cohort.

## Conclusion

This study will be the first to determine if pediatric SLE patients show higher genetic load of SNP's compared to adult SLE population and if genetic load is associated with disease severity and damage.

## Disclosure of interest

None declared.

